# Surface Topography-Based Classification of Coefficient of Friction in Strip-Drawing Test Using Kohonen Self-Organising Maps

**DOI:** 10.3390/ma18133171

**Published:** 2025-07-04

**Authors:** Krzysztof Szwajka, Tomasz Trzepieciński, Marek Szewczyk, Joanna Zielińska-Szwajka, Ján Slota

**Affiliations:** 1 Department of Integrated Design and Tribology Systems, Faculty of Mechanics and Technology, Rzeszów University of Technology, ul. Kwiatkowskiego 4, 37-450 Stalowa Wola, Poland; m.szewczyk@prz.edu.pl; 2 Department of Manufacturing Processes and Production Engineering, Faculty of Mechanical Engineering and Aeronautics, Rzeszów University of Technology, al. Powstańców Warszawy 8, 35-959 Rzeszów, Poland; tomtrz@prz.edu.pl; 3 Department of Component Manufacturing and Production Organization, Faculty of Mechanics and Technology, Rzeszów University of Technology, ul. Kwiatkowskiego 4, 37-450 Stalowa Wola, Poland; j.zielinska@prz.edu.pl; 4 Institute of Technology and Materials Engineering, Technical University of Košice, Mäsiarska 74, 040 01 Košice, Slovakia; jan.slota@tuke.sk

**Keywords:** deep drawing, friction, sheet metal forming, steel sheet, surface roughness

## Abstract

One of the important parameters of the sheet metal forming process is the coefficient of friction (CoF). Therefore, monitoring the friction coefficient value is essential to ensure product quality, increase productivity, reduce environmental impact, and avoid product defects. Conventional CoF monitoring techniques pose a number of problems, including the difficulty in identifying the features of force signals that are sensitive to the variation in the coefficient of friction. To overcome these difficulties, this paper proposes a new approach to apply unsupervised artificial intelligence techniques with unbalanced data to classify the CoF of DP780 (HCT780X acc. to EN 10346:2015 standard) steel sheets in strip-drawing tests. During sheet metal forming (SMF), the CoF changes owing to the evolution of the contact conditions at the tool–sheet metal interface. The surface topography, the contact loads, and the material behaviour affect the phenomena in the contact zone. Therefore, classification is required to identify possible disturbances in the friction process causing the change in the CoF, based on the analysis of the friction process parameters and the change in the sheet metal’s surface roughness. The Kohonen self-organising map (SOM) was created based on the surface topography parameters collected and used for CoF classification. The CoF determinations were performed in the strip-drawing test under different lubrication conditions, contact pressures, and sliding speeds. The results showed that it is possible to classify the CoF using an SOM for unbalanced data, using only the surface roughness parameter Sq and selected friction test parameters, with a classification accuracy of up to 98%.

## 1. Introduction

Deep-drawing processes are commonly used for forming sheet metal components. In a forming process, the sheet metal is subjected to large unit pressures and undergoes large deformations that change the topography of the sheet metal surface and its mechanical properties as a result of the work hardening phenomenon [1,2]. The phenomenon that allows for the control of the forming process is friction [3]. By providing appropriate lubrication adapted to the contact pressures and sliding speeds prevailing in the contact zone, the adverse effect of the friction on the forming process can be limited [4]. Excessively high friction increases the forming forces and can lead to premature cracking of the sheet metal material and accelerated tool wear [5,6]. The friction phenomenon is particularly important in the blankholder area in sheet metal forming [7]. In this area, the sheet metal is subjected to a triaxial state of stress, which leads to an intensive change in the topography of the sheet metal and friction conditions [8]. The basic way to reduce friction in deep-drawing processes is lubrication of the contact surface with liquid or solid lubricants [9]. Currently, synthetic oils are the basic lubricants in sheet metal forming, and typical oils used in other industries include engine oils, gear oils, and machine oils. Special oils for deep-drawing operations in conventional sheet metal forming processes and incremental sheet-forming techniques [10,11] are used in forming drawpieces with complex shapes from difficult-to-form materials. The lubricant should also be adapted to the processing temperature [12]. In parallel, the possibilities of using oils modified with nanoparticles [13] and bio-based lubricants [14,15] are being investigated to better adapt the type of lubricant to the forming process conditions.

Designing the technological process of sheet metal forming requires knowledge of friction and, more precisely, the coefficient of friction (CoF), which is a quantitative indicator representing the relationship between the normal force and the friction force. The strip-drawing test with flat dies is a basic tribological test for testing sheets under conditions of variable contact pressures, variable sliding speeds, and various lubrication conditions [16,17]. Its advantage is the simplicity of determining the coefficient of friction. Over the years, the strip-drawing test has been successfully used by many authors to test steel, aluminium, and titanium sheets and their alloys, in order to investigate the effects of sliding speed [18], nominal pressures [19,20], surface roughness of tools [21], tool coatings [22], and lubrication conditions [23] on the quality of the sheet surface after forming and the value of the CoF. Trzepieciński et al. [24] investigated the effect of the type of tool coating on the value of the coefficient of friction. It was found that the Ti-coated countersample, after electron pulse irradiation and magnetron sputtering, is desirable due to the effective reduction in friction. Fejkiel and Goleń [8] conducted numerical simulations of strip-drawing tests and found that the real contact area increases with the increase in the mean roughness (Ra) of the sheet.

Owing to the synergistic influence of many factors, friction is not easy to analyse. Therefore, artificial intelligence methods, including artificial neural networks (ANNs), genetic algorithms (GAs), machine learning (ML), Siamese networks, deep learning, and other analytical methods (analysis of variance, response surface methodology) have engendered interest among tribologists in analysing the friction phenomenon. Trzepieciński and Szpunar [25] used a radial basis function ANN to evaluate the influence of lubrication conditions and contact pressure on the CoF of Ti-6Al-4V sheets. The results allowed for the determination of the relationship between contact pressure and CoF. Rahardja et al. [26] developed an ML model, optimised by a GA, to characterise the tribological behaviour of CuZr metallic glasses. The results indicated that the CoF could be predicted with a determination coefficient of R^2^ = 0.95. Baş and Karabacak [27] investigated the effects of bearing load and temperature on the CoF by means of an ML algorithm. It was found that support vector machine and regression trees algorithms can successfully predict the variation in CoF. Najm et al. [28] applied the Gradient-Boosted Regression Trees algorithm to find the influence of the friction test parameters on the coefficient of friction in the drawbead area in sheet metal forming. The ability of the model to predict the CoF value, determined by the coefficient of determination, was equal to R^2^ = 0.972. The role of ANNs and machine learning in combining tribological experimental data and analytical methods is presented in [29,30].

Conventional friction monitoring techniques encounter a number of problems related to, among other things, the specialist requirement for supervising the model training and the difficulty of identifying features sensitive to changes in the CoF. To overcome these difficulties, self-organising maps (SOMs) can be used. Kohonen networks (SOMs) are a type of artificial neural network performing unsupervised learning. This means that during training, no output patterns are presented for the input data. The network’s task is to create such patterns during the training. The training data are independently classified by the network based on their mutual correlation. Brito et al. [31] used an SOM to identify tools’ frictional wear in turning. It was found that it is possible to predict the cutting tools’ wear condition using only the vibration signal, with up to 92% accuracy. The use of SOMs to identify the friction conditions in sheet metal forming processes is very limited. Zhang et al. [32] used the Response Surface Classification Study Method to establish the friction maps for Ag/MoS_2_/WS_2_ nanocomposites under different friction conditions. It was found that the higher sliding speed and the higher normal force resulted in the oxidation delamination of the sample’s surface.

Recently, the state of ultralow friction between two sliding surfaces, i.e., superlubricity, has become a pioneering topic in tribology. In [33], a review of the findings of superlubricity fluid materials from five different categories is presented to show the contributions of hydrodynamics and hydration to superlubricity fluids. Furthermore, in this paper, the uniform mechanisms of superlubricity fluids are summarised for various liquid lubricants in boundary, mixed, and hydrodynamic lubrication regimes.

To the best of the authors’ knowledge, this paper presents for the first time the application of an SOM to monitor the friction phenomenon in sheet metal forming. To overcome the difficulty in identifying the signal parameters used to determine the CoF, this paper proposes a new approach to apply unsupervised artificial intelligence techniques with unbalanced data to classify the coefficient of friction of DP780 (HCT780X acc. to EN 10346:2015 standard [34]) steel sheets in strip-drawing tests. The SOM was created based on the surface topography parameters collected during each friction test.

## 2. Materials and Methods

### 2.1. Materials

The test material in the study was a 1 mm thick dual-phase (DP) DP780 steel sheet. The chemical composition of this steel is presented in Table 1. Selected mechanical properties were determined in accordance with the EN ISO 6892-1:2020 standard [35], using a uniaxial tensile testing machine (TIRAtest 2300, TIRA Maschinenbau GmbH, Rauenstein, Germany). The samples for the friction tests and tensile tests were cut along the rolling direction of the sheet metal. The tests were carried out with five repetitions, which allowed for the determination of the average values of the selected mechanical parameters (Table 2). DP steels are used in the automotive industry and are distinguished by a unique combination of high plasticity, high tensile strength, and susceptibility to cold working. Significant work hardening at the initial stage of deformation allows DP steel to effectively prevent local necking due to effective stress redistribution, which ensures uniform elongation of the material. The samples for the friction tests were prepared in the form of strips of 20 mm (width) × 135 mm (length) × 1.0 mm (thickness).

**Figure 1:**
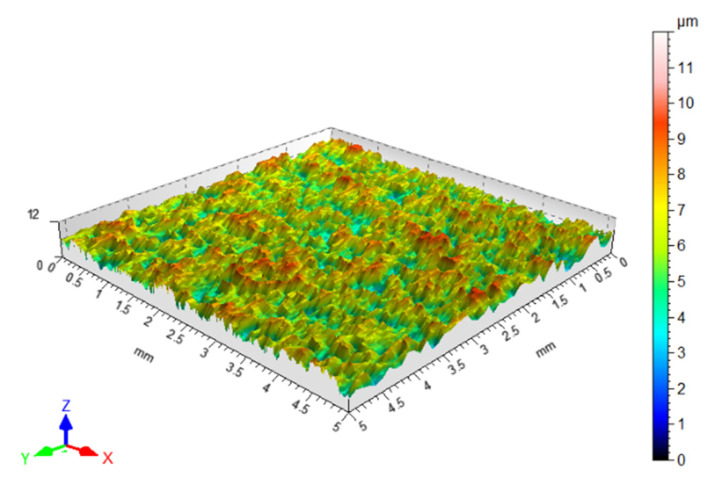
Surface topography view of DP780 steel sheet in as-received state.

The surface quality of the sheet metal in the as-received state was assessed by measuring the surface topography (Figure 1) using a Hommel-Etamic T8000RC stationary profilometer from Jenoptik AG (Jena, Germany). Basic surface topography parameters (Table 3) were determined according to the ISO 25178-2:2022 standard [36]. The surface roughness measurement conditions are listed in Table 4.

For the analysed parameters, calculations were performed based on data from a square surface of 5 mm × 5 mm. The step between measured points in a line was 0.5 μm, while the step between individual lines was 0.1 mm. The roughness parameters were measured on one (always the same) side of the sheet metal strips.

### 2.2. Experiment

**Figure 2:**
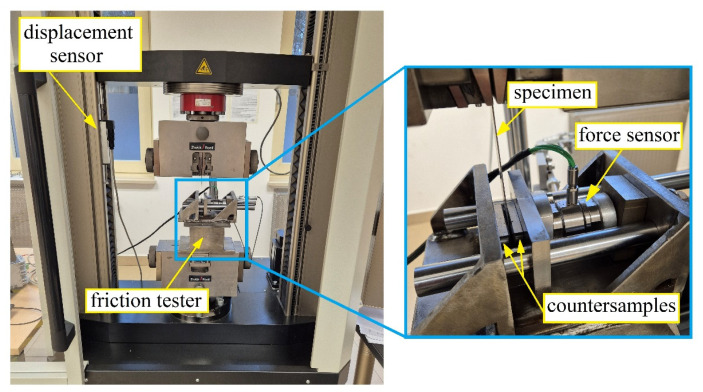
Friction test stand.

The strip-drawing tests were carried out using a specially designed tester mounted in the lower holder of a Zwick/Roell Z100 testing machine (Zwick Roell Group, Ulm, Germany) (Figure 2). The strip samples were pulled between the two flat countersamples at a specific contact pressure. The friction force was recorded using the testing machine’s measuring system, while the normal force was measured using a Kistler 9345B piezoelectric force sensor (Kistler, Winterthur, Switzerland) and a program developed in the Labview environment. When acquiring the force signals, the sampling frequency was 100 Hz. The values of both the pulling and normal forces were synchronised in the time domain using an original application created in the Labview environment. A Megatron Series SPR18 displacement sensor (Megatron Elektronik GmbH & Co. KG, Putzbrunn, Germany) was used to measure the sample displacement.

**Figure 3:**
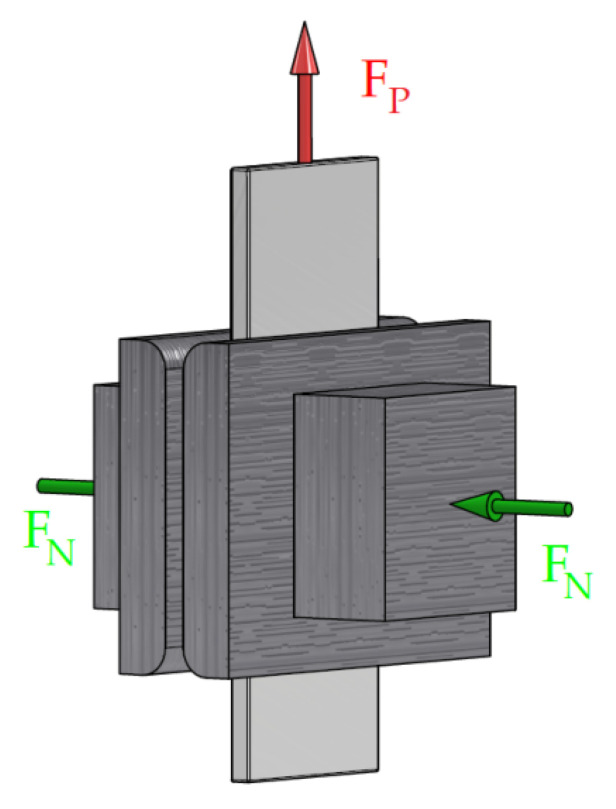
Schematic diagram of the strip-drawing test.

The test procedure consisted of drawing a strip-shaped sample between two countersamples with flat working surfaces (Figure 3). The countersamples were made of 145Cr6 cold-worked tool steel, which is characterised by high wear resistance. The friction test allowed for the accurate reproduction of the surface contact conditions in the blankholder area in sheet metal forming and allowed for the precise determination of the CoF value based on the values of the pulling force (F_P_) and the normal force (F_N_).

The values of the CoF were calculated based on the obtained measurement signals concerning the normal force F_N_ and the corresponding pulling force F_P_. The relationship presented in Equation (1) was used to determine the coefficient of friction, as follows:

(1) $$CoF=\frac{F_{P}}{2\;F_{N}}$$

Oils commonly used as lubricants in deep-drawing operations were used in the tests. Immediately before each test, the lubricant was distributed uniformly on the surface of the samples at 2 g/m^2^, using a shaft. The kinematic viscosity of these oils, according to the manufacturer’s data, is listed in Table 5. The experiments were carried out at nominal contact pressure values p_N_, defined as the ratio of the contact (normal) force to the contact area of the sheet with the countersamples. Nominal contact pressures of 2, 6, 10, and 14 MPa and various sliding speeds of 30, 60, and 90 mm/min were used in the friction tests. The research attempted to reproduce industrial forming conditions, so sheets in the as-received state were used in the friction tests. Table 6 presents the sets of strip-drawing parameters used in the tests.

**Figure 4:**
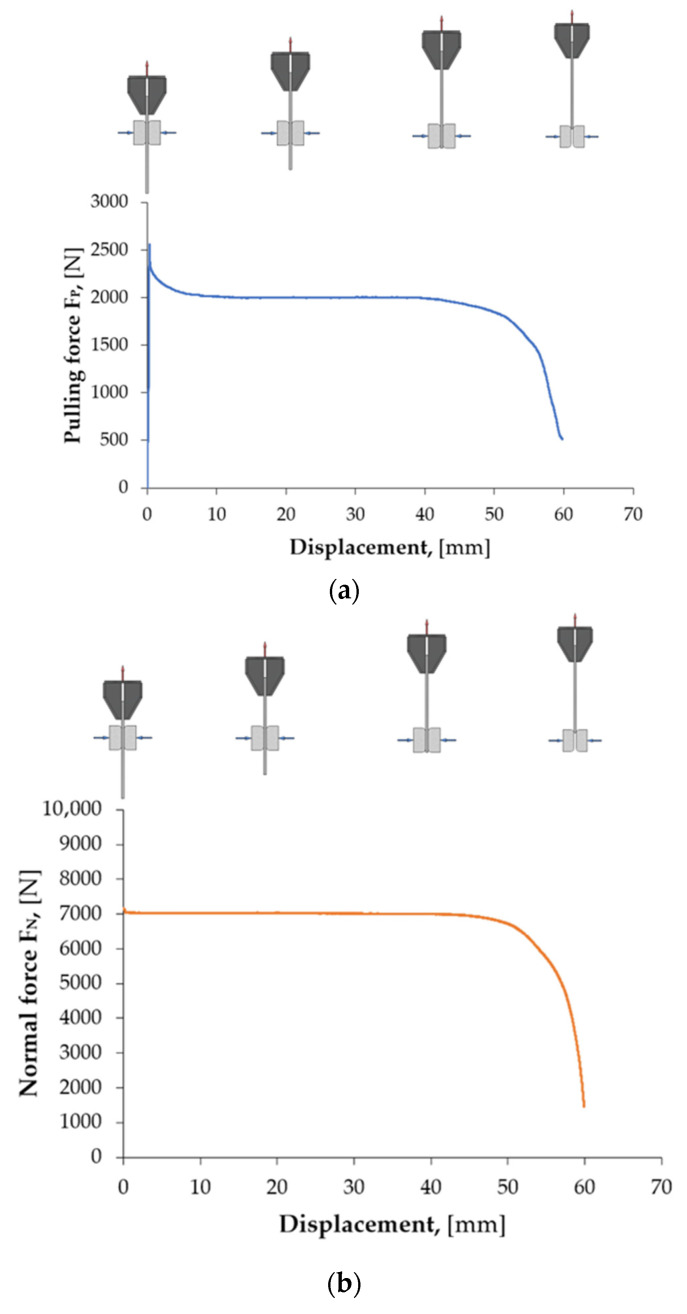
Change in (**a**) pulling force and (**b**) normal force during the friction process under the following conditions: normal force F_N_ = 7000 N, sliding speed 30 mm/min, lubrication with S100 Plus oil.

Figure 4a shows an example of the variation in the pulling force signal recorded using the measuring system of the testing machine during the friction test, with the normal force F_N_ = 7000 N (Figure 4b). Characteristic sections can be distinguished in the recorded pulling force course, depending on the displacement of the sheet metal strip relative to the countersamples. At the beginning of the strip-drawing test (for a sheet metal strip displacement between 0 mm and about 0.3 mm), a rapid increase in the pulling force was observed. This is related to overcoming the static friction, in which the coefficient of friction reaches higher values compared to kinetic friction. When the contacted bodies start to move relative to each other, the pulling force value decreases, the value of which stabilises after a short displacement. The pulling force remains at a stable level until the drawn sheet metal strip begins to leave the area of the countersamples (sheet metal strip displacement of about 45 mm). Then, the pulling force and normal force values quickly decrease.

## 3. Force Signal Acquisition

Based on the recorded signals of the pulling and normal forces, the CoF value was determined (Equation (1)), based on the average of the recorded values of the force signals from the individual signal fragments. It is not possible to automatically measure the CoF on the entire (continuous) courses of the force signals (F_P_, F_N_) from the sensors, because they may contain a number of disturbances. The presence of disturbances limits the reliability of the CoF value determined from the entire friction test.

To overcome this limitation, it is necessary to develop a procedure that allows for the selection of signal fragments that are not burdened with disturbances. In most scientific studies, manual selection of signal fragments is commonly performed by marking the interesting signal fragment or using simple algorithms that check whether the signal has exceeded a predetermined threshold. The best solution is to use an additional program that divides the signal into fragments called segments. Such a program evaluates the quality of the selected segments and allows for the analysis of only those that are suitable for determining the CoF value.

**Figure 5:**
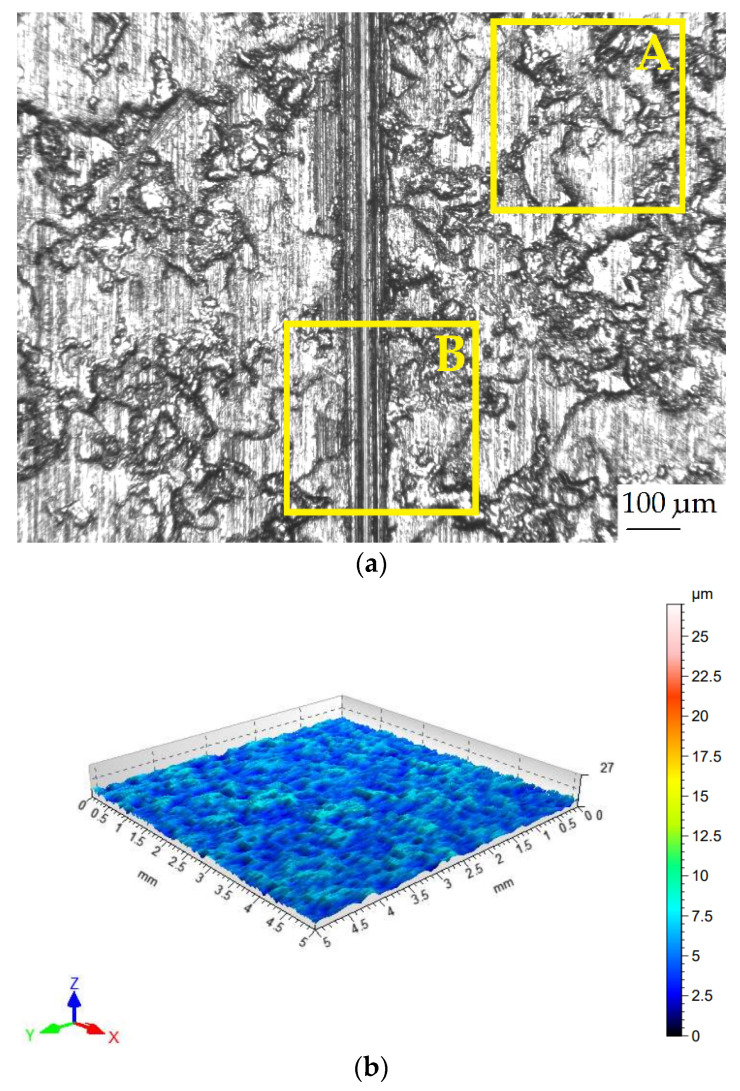

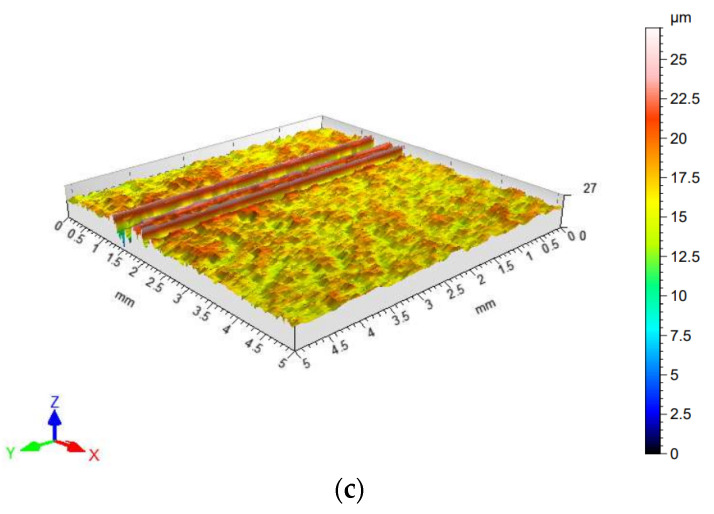
(**a**) Optical micrograph of sheet surface after friction test with a disturbance in the form of a scratch, and surface topographies of areas (**b**) A and (**c**) B marked in subfigure (**a**); subfigures (**a**–**c**) concern the sheet surface after a friction test at a contact pressure of 2 MPa, sliding speed of 60 mm/min, and with no lubrication.

Segmentation consists of dividing the signal during its acquisition into equal fragments in the time domain, e.g., 5 s long. Based on such segments, the average value of the forces is determined, based on which the CoF is estimated. However, before this procedure, it is necessary to select such segments that will best represent the strip-drawing test. Segments of stable signals are the most desirable. This allows for avoiding random changes in the signal, resulting, for example, from the sheet metal leaving the countersamples’ area, or from accidental scratches on the surface of the test sample. Figure 5 shows an example of local scratching of the sheet metal strip as a result of friction process. Such local scratches generate fluctuations (changes in value) in the force signals, which lead to an incorrect determination of the CoF value. Therefore, it was decided to develop a methodology for determining the CoF value based on Equation (1), which allows for accounting for the disturbances that occur.

**Figure 6:**
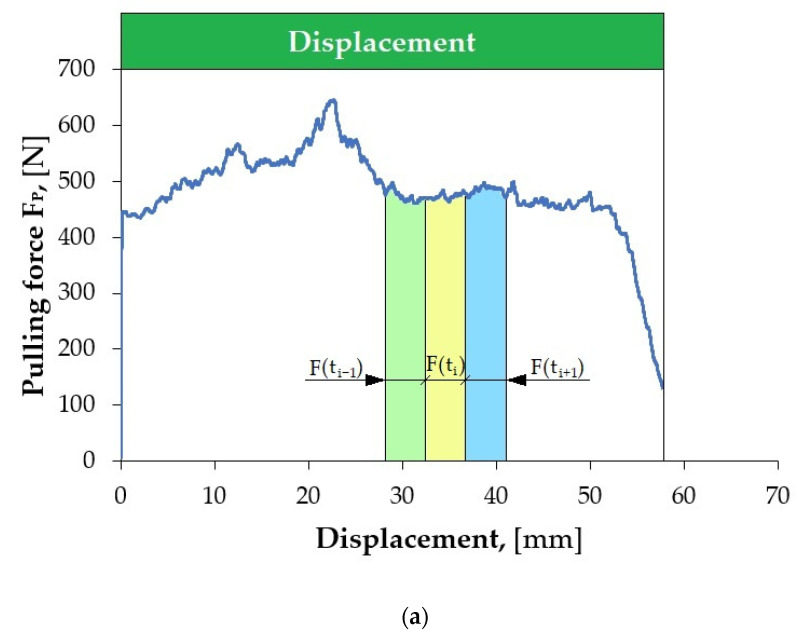

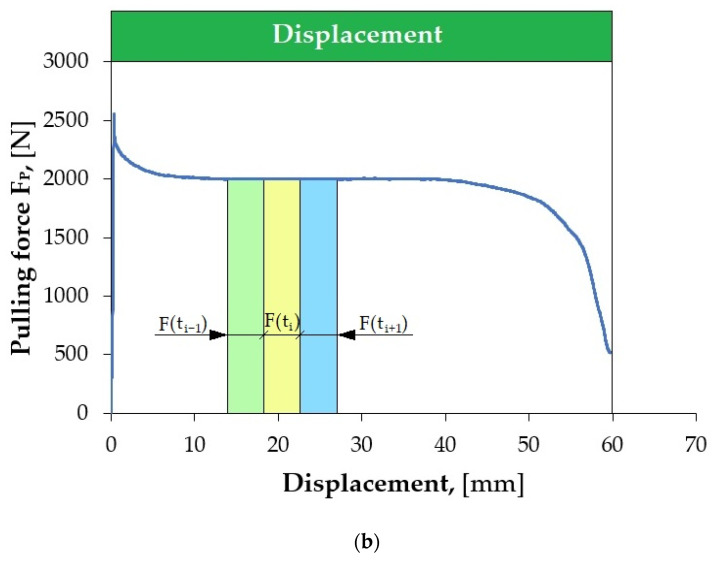
(**a**) Disturbed and (**b**) non-disturbed variations in F_P_ force signal in the strip-drawing test under the following conditions: (**a**) normal force 1000 N, sliding speed 60 mm/min, dry friction; and (**b**) normal force 7000 N, sliding speed 30 mm/min, lubrication with S100 Plus oil.

**Figure 7:**
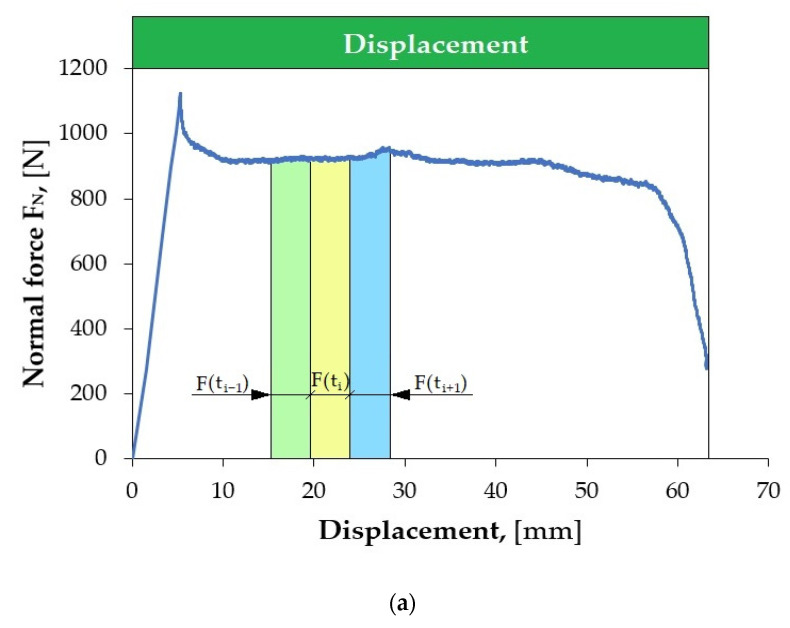

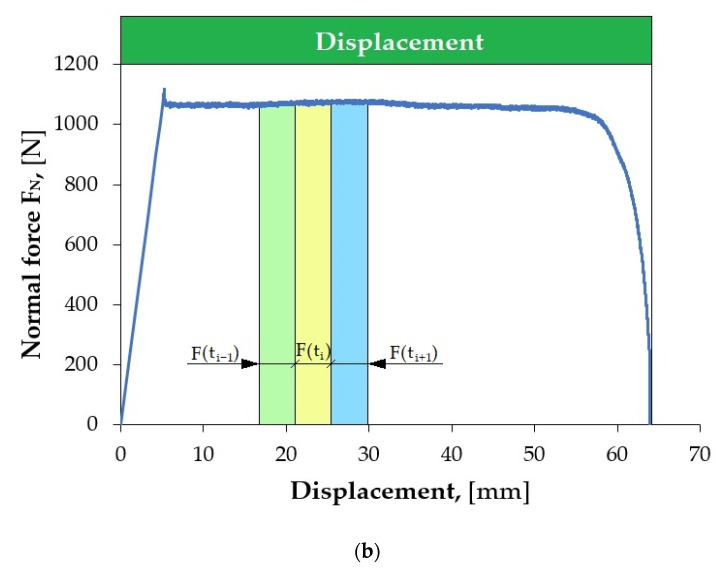
(**a**) Disturbed and (**b**) non-disturbed variations in F_N_ force signal in the strip-drawing test under the following conditions: (**a**) normal force 1000 N, sliding speed 30 mm/min, with dry friction; and (**b**) normal force 1000 N, sliding speed 60 mm/min, with dry friction.

The methodology of signal (F_Fl_) invariance evaluation is presented in Figure 6 and Figure 7, and it is described by the following relation:

(2) $$F_{\mathrm{Fl}}=\left|\frac{\left[F(t_{i-1})\right]}{\left[F(t_{i})\right]}\;-\;1\right|+\left|\frac{\left[F(t_{i})\right]}{\left[F(t_{i+1})\right]}\;-\;1\right|\;$$

The lower F_Fl_ is, the more suitable the segment is for determining the CoF. In order to select the best fragments of the force signal, their uniform distribution in the displacement domain was maintained.

It is not possible to automatically evaluate the CoF on entire (continuous) signal courses from force sensors, as they may contain a number of random disturbances. It is necessary to automatically select appropriate fragments of force signals from the friction test. In most analyses, manual selection of signal fragments is commonly performed by marking the interesting fragment of the signal or using simple algorithms that check whether the force signal exceeds a predetermined threshold. The best solution is to use an additional program that divides the force signal into fragments called segments, and then another one that evaluates the quality of the selected segments and allows for analysis of only those that will be related to the friction process. Segmentation therefore consists of dividing the signal into equal fragments during its acquisition, e.g., 1 s long. From such segments, measures are generated (e.g., average value, root-mean-square deviation), based on which the value of the CoF is estimated. However, before these procedures can be carried out, it is necessary to select such segments that will best represent the strip-drawing test. The most suitable signal fragments come from its unchanging parts. This allows for avoiding random changes in the force signal resulting from process disturbances. The lower F_Fl_ is, the more suitable the segment is for determining the CoF value.

The first limitation applied was to set the maximum number of segments in the friction process. Their selection was made after the strip-drawing test process was completed. The described method of segmentation allows for selecting appropriate segments of the recorded force signals from subsequent force variation courses. After selecting a given segment, the average value of a specific signal is determined from each available measurement channel, and the original force signal courses are deleted, saving computer memory. The CoF value is estimated independently for each segment.

## 4. Analysis of Variance

The study of the influence of the parameters of the friction process (nominal pressure, oil viscosity, sliding speed) on the value of the CoF began with the use of analysis of variance (ANOVA). This analysis allowed for the determination of the significance of the influence of individual strip-drawing test parameters on the value of the CoF. The significance of the influence of the selected parameters on the CoF was assessed at the significance level of *p* ≤ 0.05. Detailed results regarding the significance of these parameters are presented in Table 7.

Based on the *p*-value (Table 7), it was found that the key factors that have a statistically significant effect on the CoF are the lubrication conditions and nominal pressure. On the other hand, the significance level obtained for the sliding speed indicates that this parameter does not have a significant effect on the value of the CoF.

**Figure 8:**
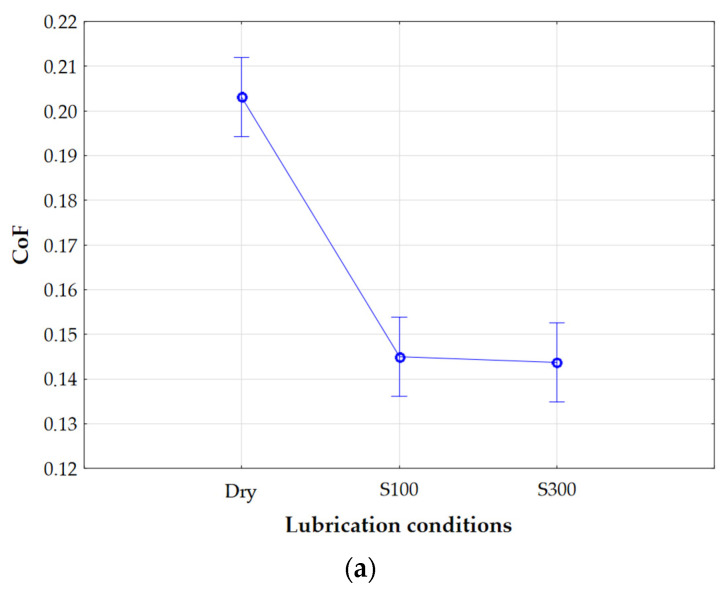

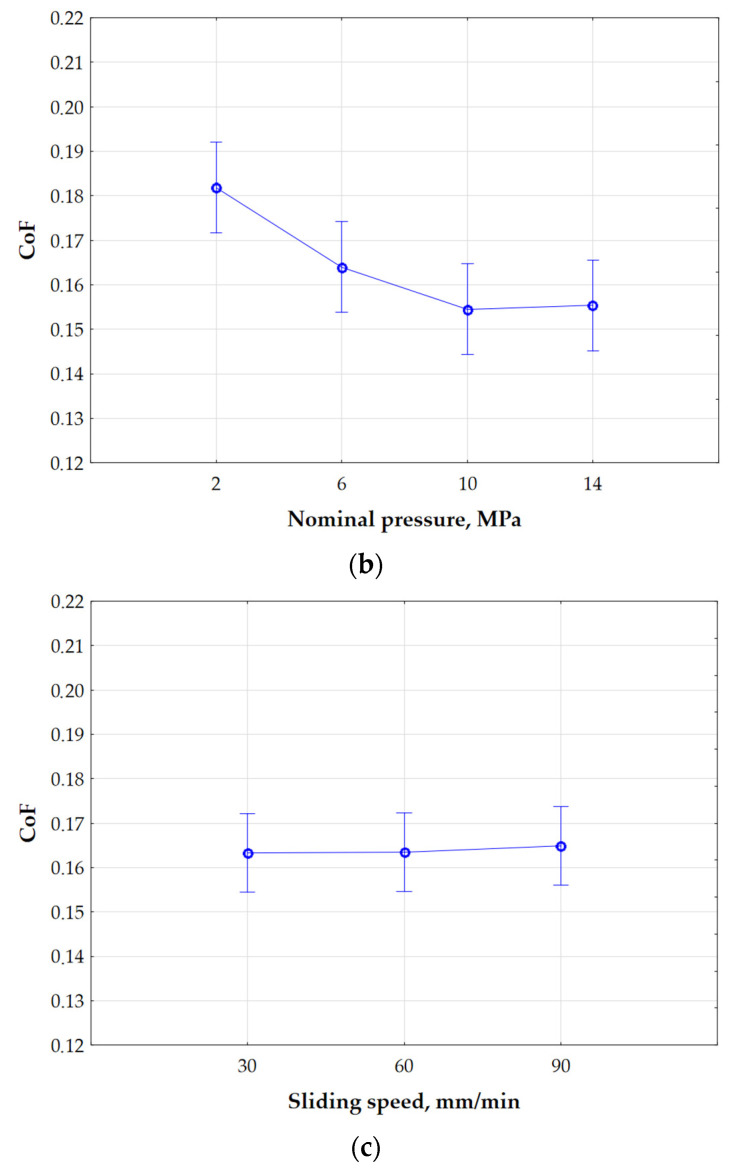
Effects of individual process parameters on the CoF: (**a**) lubrication conditions, (**b**) nominal pressure, and (**c**) sliding speed.

The influence of the individual process parameters on the CoF was presented in graphs illustrating the dependence between the CoF value and the lubrication conditions (Figure 8a), nominal pressure (Figure 8b), and sliding speed (Figure 8c). Additionally, vertical lines (95% confidence interval) were marked on the graphs in places corresponding to the parameter values used in the tests. When analysing the effect of one input on one output in a multivariate analysis of variance, the other input parameters are held at their average values. This is a typical approach used in analysis-of-variance software. In the case of the graph showing the dependence of the CoF on the lubrication conditions, the absence of lubricant results in significantly higher CoF values. In contrast, in the analysis of, for example, the dependence of the CoF on the nominal pressure (e.g., for the value of 2 MPa), data from both tests conducted under dry friction conditions and with the use of different lubricants are included. As a result, the averaged CoF values for a given nominal pressure are lower, which is a consequence of the analysis of variance limited to one parameter (nominal pressure) while varying the other conditions, including the lubrication conditions.

## 5. Kohonen Self-Organising Map

The Kohonen network was used to find clusters with similar features. It is a type of self-organising network that has two layers—input and output—which represent (after training the network) a topological map. The input vector X, describing certain characteristic features of phenomena occurring in the environment, is subject to normalisation according to

(3) $$x_{\mathrm{zj}}=\frac{x_{j}}{\sqrt{\sum_{k-1}^{n}{{(x}_{k})}^{2}}}$$

where x_zj_—normalised variable, x_j_—variable subject to normalisation, and x_k_—other data values of the analysed input variable.

The normalised information is translated into stimuli that excite neurons as an N-dimensional signal X from a set of training patterns. The synaptic connections of neurons store weights (of the same dimension as the input vector) in the form of a vector Wi = [w_i1_, w_i2_, …, w_in_]. The calculations are started for weights equal to small random numbers, taking values from the interval (0, 1). During training, these weights are modified in such a way as to best reflect the internal structure of the input data. Neurons compete with each other, and the winner is the one whose weights differ the least from the corresponding components of the input vector. The w-th neuron (winner) satisfies the relation

(4) $$d(x,\;w_{w})=\underset{i=1,2,\ldots ,n}{\min}d(x,w_{i})$$

where d(x, w_w_)—distance in the sense of the selected metric between vector X and vector W, and n—number of neurons.

The most commonly used measure of the distance between the input vector X and the weight vector W_i_ is the Euclidean measure:

(5) $$d\left(x,\;w_{i}\right)=\left\|x-w_{i}\right\|=\sqrt{\sum_{j=1}^{N}{\left(x_{j}-w_{\mathrm{ij}}\right)}^{2}}$$

Around the winning neuron, a topological neighbourhood S_w_(n) is assumed, the radius of which decreases during the computation. The neighbourhood should be understood as the set of neurons located around the winning neuron. Then, the winning neuron and its neighbouring neurons are subject to adaptation according to the Kohonen rule:

(6) $$w_{i}\left(n+1\right)=w_{i}\left(n\right)+\propto_{i}\left(n\right)[x-w_{i}\left(n\right)]$$

where α_i_(n) is the coefficient of the i-th neuron from the neighbourhood S_w_(n) in the k-th learning step. This coefficient is called the learning coefficient, takes values from the range (0, 1), and its value decreases to 0 with the distance from the winner. The weights of neurons outside the adopted neighbourhood are not subject to changes.

Based on the results from the ANOVA, the feature describing the sliding speed of the sheet metal strip was omitted in further studies and in the modelling process of the Kohonen network. The SOM is an unsupervised learning algorithm, which means that it does not require labelling of data [39]. Instead, the network itself detects dependencies in the data, which makes it a particularly useful tool in data exploration. The operation of this network is based on the competitive learning mechanism, in which neurons compete for the ability to represent the input vectors provided to them [40,41]. In practice, this means that in each iteration of the learning process, individual neurons analyzse the input data, and then the one that best matches the given pattern is selected as the winning neuron. Its weights, as well as the weights of neurons in its neighbourhood, are then updated towards the provided vector, which over time leads to the creation of an ordered data structure. One of the main applications of the Kohonen network is cluster analysis (clustering), that is, grouping similar data into sets with similar properties [42].

**Figure 9:**
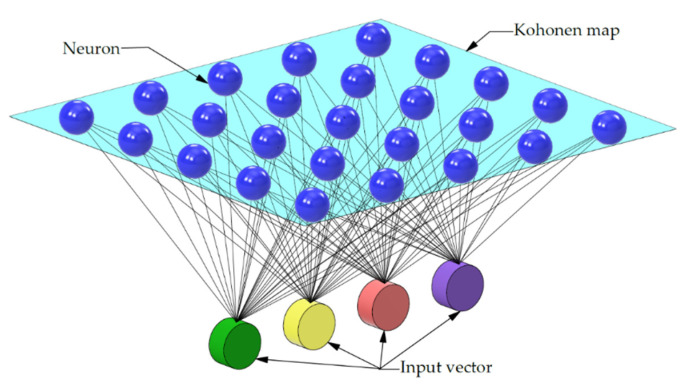
A Kohonen network with a 5 × 5 output graph.

Figure 9 shows an example of a two-dimensional Kohonen map, consisting of 25 neurons arranged in a 5 × 5 grid. This structure is designed to allow for mapping and organising the features of the provided input dataset. Each neuron acts as a representative of a certain fragment of the data space, and its weights are gradually adjusted during the learning process, which allows for gradual grouping of similar patterns in neighbouring areas of the grid.

Training the Kohonen network involves assigning cluster centres to a layer of radial neurons. During training, the algorithm finds the neuron whose centre is closest to a given training case. This neuron and its neighbours are matched so that they are more similar to the current case. Before starting to build the Kohonen network, the data were subjected to the process of normalisation to the range from 0 to 1. For this purpose, min/max normalisation was used, using Equation (7):

(7) $$X=\frac{x-x_{min}}{x_{max}-x_{min}}$$

The process of building the Kohonen network began with determining the quantitative input variables of the network. When determining the quantitative inputs, variables such as lubrication conditions, nominal pressure and one of the surface roughness parameters (Sq, Ssk, Sku, Sp, Sv, Sz, and Sa) were considered. In each analysis using the Kohonen network, only one of the mentioned roughness parameters was used as input. When taken into account, the above variables allowed for the preparation of a set of input data that could be used to effectively train the Kohonen network. The lubrication conditions were classified based on the kinematic viscosity of the lubricant used, by selecting the following values: 0 for dry friction, 360 mm^2^/s for lubrication with S100 Plus oil, and 1135 mm^2^/s for lubrication with S300 oil. The next parameter taken into account was the nominal pressure, assuming four defined levels in the tests, i.e., 2, 6, 10, and 14 MPa. This enabled the analysis of the influence of different loads on the behaviour of the tribological system. All of the parameters were normalised using min/max normalisation (Equation (7)).

The measure of the quality of a neural network’s performance is the degree of generalisation of knowledge contained in the training data as the ability to predict the class for new data not used in the training process. The problem of knowledge generalisation is one of the most important issues in network training [43]. It is quite easy to train an appropriately complex network to accurately adapt to the training data, but the network will then contain the noise contained in the training data, which may reduce its ability to predict outcomes for new input data. This is the problem of overfitting, which must not be allowed. One way to protect the ANN against overfitting is to artificially separate a subset that will not be used to train the network, and which can be used to check whether the network has generalised the knowledge obtained from the training data well. It may even be useful to separate three subsets: training, test, and validation [28,44]. The first is used to train the network, the second to control the training process, and the third for the final verification and selection of the best network.

**Figure 10:**
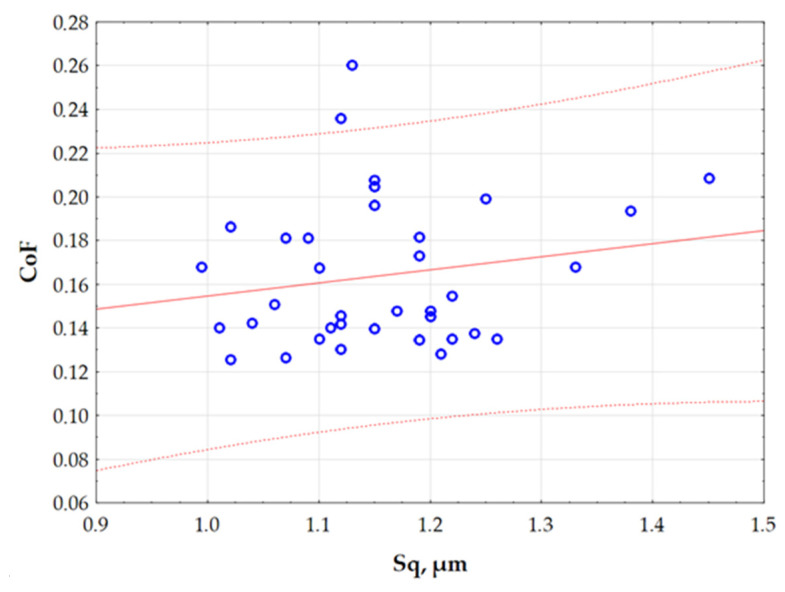
Interrelation between the Sq parameter and CoF.

The assignment of data cases to individual subsets in the Kohonen network could be performed randomly or by using an appropriate variable containing two or three different values that inform about the cases belonging to the subset. Then, the sampling methods were determined, indicating the division of the random sample size into 70% for training samples and 15% each for testing and validation. As a result of the random division of the experimental dataset, 26 samples were assigned to the training set. The remaining data were divided evenly: five samples to the test set and five samples to the validation set. Random data sampling allowed for the avoidance of biases resulting from the order of samples or their previous grouping. In order to ensure the appropriate data distribution and increase the efficiency of the training process, a random initialisation of the neural network based on the Gaussian distribution was used. The determined dimensions of the topological map (output layer of the network) created a square, 2 × 2 grid of neurons. The established proportions of the map sizes and the number of nodes had an impact on the quality of network training. The surface topography parameter Sq was used as the final component of the input vector. Figure 10 shows the relationship between two quantitative variables (Sq and CoF), with a confidence interval of 95%. The graph helps to identify potential correlations between variables, and to detect clusters of observations. The values of the variables used to create the graph in Figure 10 were not normalised. It can be observed that the relationship between the parameters Sq and CoF described by the linear function divides the range of variation in the Sq parameter into clear two intervals. For this reason, the division of the CoF variation into two ranges was adopted. Therefore, a two-state classification was adopted, in which an object or phenomenon can be assigned to one of two possible categorical states. This is a simple and commonly used way of organising data, especially in the context of binary classification.

The variation in CoF values was divided into two ranges: ‘low coefficient of friction—LCF’ (between 0 and 0.4) and ‘high coefficient of friction—HCF’ (between 0.4 and 1). Values listed in brackets correspond to normalised values (Table 8). In this study, because the classification problem is related to two conditions of CoF values, four neurons were used. The first and second neurons represent a ‘low coefficient of friction’, and the third and fourth represent a ‘high coefficient of friction’. Neurons 1-1 and 1-2 were assigned to ‘high coefficient of friction’. On the other hand, the neurons 2-1 and 2-2 were assigned to ‘low coefficient of friction’. Neuron 1-1 represents the friction phenomenon in dry friction conditions, and neuron 1-2 represents high nominal pressure. Neurons 2-1 and 2-2 represent the CoF for lubrication conditions with S300 and S100 oils, respectively.

The developed network, designated as SOM3-4, describes the number of input variables (three: lubrication conditions, nominal pressure, and selected roughness parameter) and the number of generated neurons (four: network with dimensions of 2 × 2).

The Kohonen networks use an activation threshold when classifying input data. This is the required approach in classification tasks using Kohonen networks. In a classification neural network, there are two threshold values: an acceptance threshold and a rejection threshold. These parameters are used in classification and allow a decision to be made regarding the assignment of an object to a given class. Because the activation level of a neuron in a Kohonen network is equal to the distance of the neuron from the input case, the acceptance threshold is the maximum distance at which the similarity of objects is still recognisable. If the activation of the winning neuron is greater than this distance, the Kohonen network is considered to be inconclusive, and the response is undetermined. In this way, by assigning labels to all neurons, one by one, and setting the acceptance threshold value accordingly, the Kohonen network gains additional functionality. After training, the network can act as a detector, which reports indecision only if the input case is dissimilar to any of the radial neurons.

**Figure 11:**
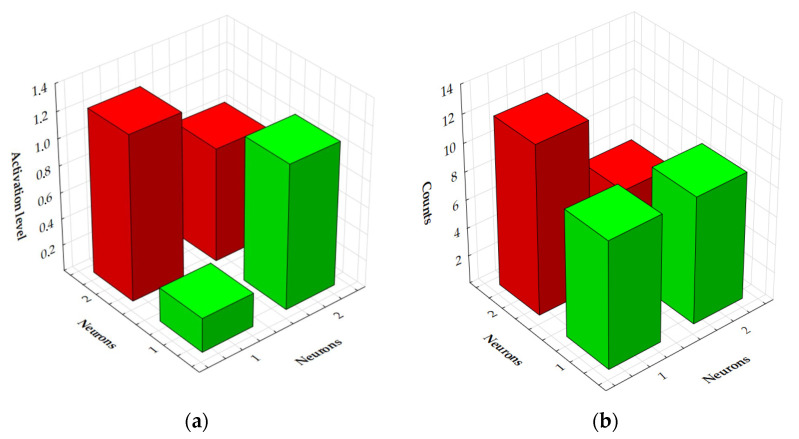
(**a**) Activation level for the SOM network and (**b**) numbers of measurements assigned to individual neurons.

In the Kohonen network, the winning neuron located in the layer creating the topological map (in the output layer) that has the highest level of activation is used for recognition. This means that the winner is the neuron that achieves the minimum value of the distance of the input case from the point represented by the neuron (or rather, by the set of its weight coefficients). In general, since the neurons included in the Kohonen topological map can be assigned labels with class names, by knowing which neuron is the winner, we also know which class was recognised. In practice, this issue is much more complex. If we require the identity of the input signal and the pattern of the recognised class stored in the form of a set of weighting coefficients of the winning neuron, then recognition would be impossible for most input signals. Meanwhile, Kohonen networks also make sensible decisions about classification for previously unknown input signal vectors. If the distance between the input vector characterising the considered case and the pattern of a certain established class is sufficiently small, then the case is classified into this class. The acceptance level parameter is mainly used, which in this case determines the largest permissible distance of the input vector and the weight vector (class pattern) that allows the considered case to be classified into the indicated class. If the case entered at the input is located further from the winning neuron than is specified by the maximum permissible distance, then such a case is not classified. Figure 11a presents individual values of the acceptance level for four neurons adopted in the Kohonen network model. Figure 11b shows the numbers of measurements assigned to individual neurons.

The activation level values for individual Kohonen network neurons were presented in numerical form using a decimal point, which allows for precise reading of the degree of stimulation of a given neuron by specific input data. Referring to the results presented in Table 9, a detailed distribution of the activation values for each of the neurons that make up the network structure is presented.

The analysis of the number of cases assigned to individual neurons shows that neuron 2-1 represents the highest frequency, which means that the largest number of samples was assigned to it among all of the neurons in the network. This indicates that this neuron represents the most common or most characteristic data pattern occurring in the analysed set. Table 10 presents detailed characteristics of the assignment of input data to specific neurons of the Kohonen network. This allows for tracking the grouping structure and performing a more in-depth analysis of the similarities between samples in the context of their representation by neurons in the space of the SOM.

## 6. CoF Classification Results

The data collected during the experiments were analysed in terms of the possibility of using selected 3D parameters of the surface roughness to classify the CoF occurring in the strip-drawing test.

**Figure 12:**
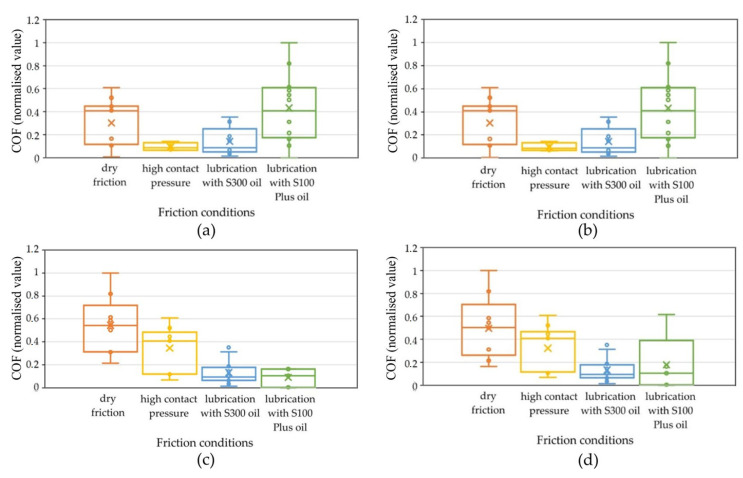
Box plot showing assignment of the CoF values to individual neurons of the Kohonen network, depending on the used variable roughness parameters: (**a**) Sa, (**b**) Sku, (**c**) Sq, and (**d**) Ssk.

The CoF classification was performed using a set of process parameters that contained the most significant information about the CoF. This was required by the diverse conditions of the friction tests. The group of features that showed the highest correlation with the CoF were selected as input data in the Kohonen model classifiers. A visual analysis of box plots of significant features affecting the CoF was performed. A box plot is a graphical tool that is used to visualise the distribution of statistical data. It is an extremely useful way to present various aspects of a dataset, e.g., its central tendency, dispersion, and possible outliers. To determine the differences in the obtained results of CoF classification resulting from the selection of input vectors to the Kohonen network in terms of their significance and unimportance, Figure 12 presents box plots for the surface roughness parameters. These graphs contain information on the position, dispersion, and shape of the CoF distribution, depending on the selected surface topography parameter, pressure, and lubricant type. Figure 12 shows the results of assigning the CoF value to the appropriate neuron by the Kohonen network, depending on the selected surface topography parameter at the input to the network. Orange corresponds to neuron 1-1, yellow corresponds to neuron 1-2, blue corresponds to neuron 2-1, and green corresponds to neuron 2-2.

The analysis carried out using the self-organising Kohonen network took into account a number of variables, including lubrication conditions, nominal pressure, and selected parameters describing surface roughness, of which the Sq parameter was finally focused on. However, at the initial stage of the research, the full set of surface roughness parameters was taken into account, as well as the sample sliding speed. Only after the initial analysis of the results was the set of analysed variables narrowed down. Based on the results of the Kohonen network, it was noted that the Sq parameter best reflects the relationship occurring between the data, especially in the context of the division of neurons in relation to the CoF value. Clear differences in the activation of neurons were detected depending on the value of the Sq parameter (Figure 12c). Sq was the most significant parameter from the set of input vectors.

Figure 12c shows a wide distribution of the CoF population variation in the case of dry friction with the use of different contact pressure values. The median CoF is 0.55. In the next case, where high pressure values and lubrication were used, a smaller distribution of the CoF population was observed. The median of the distribution falls at about 0.4 CoF. In the case of using S300 and S100 oils, the median is about 0.1 CoF. A clear trend of the median CoF value for the considered cases can also be observed, which can be described by a rational function.

The box plot analysis was used to find which features were most suitable for use as criteria for identifying the CoF. A box plot is useful for comparing the range and distribution of groups of numerical data. An illustration of the data is a box with whiskers on the side and a centre line inside. The whiskers show the maximum and minimum reference values used to exclude outliers. For the Sq parameter, the lower quartile is 0.33; 25% of all CoF values fall below this value, and 75% above it, while 25% of the data fall above the upper quartile. The interquartile range includes 50% of the data. In a box plot, the solid line indicates the median. The T-shaped whiskers go to the last point, which is still within 1.5 times the interquartile range.

**Figure 13:**
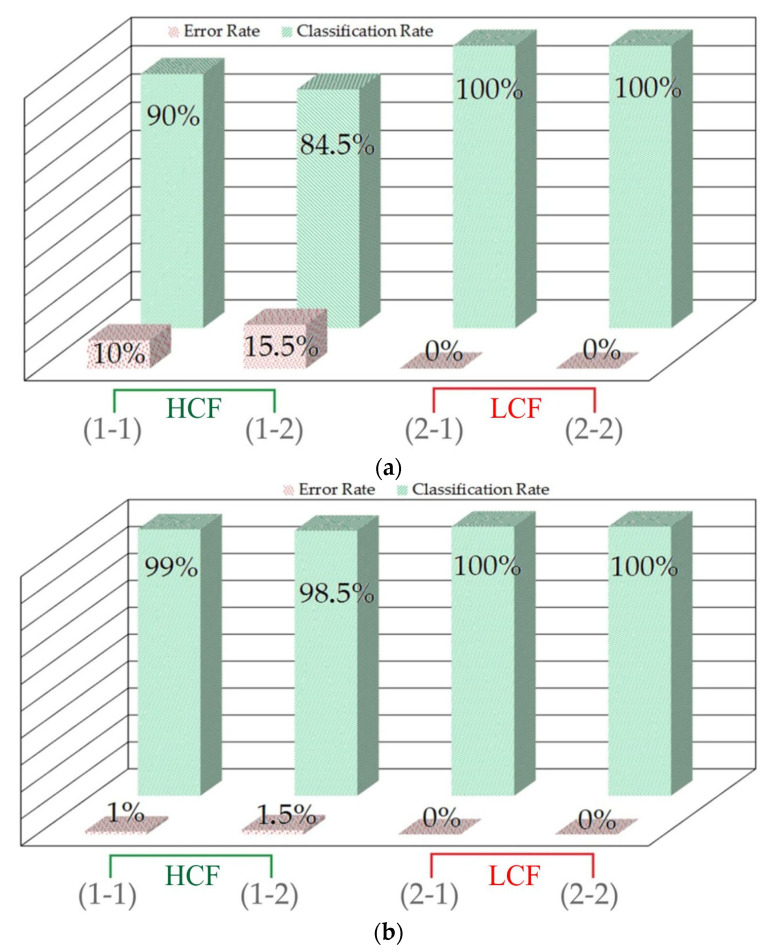
Error rate and classification rate of the SOMs classifying the coefficient of friction based on (**a**) original (unsegmented) data and (**b**) segmented data.

The tests were performed using the model configurations described in Section 5 and the following parameters: kinematic viscosity of oil, sliding speed, and nominal contact pressure. The model accuracy for validation is shown in Figure 13, where the classification rate and error rate are defined as follows:

(8) $$\mathrm{Classification}\;\mathrm{Rate}=\;\frac{\mathrm{Correctly}\;\mathrm{classified}\;\mathrm{samples}}{\mathrm{Misclassified}\;\mathrm{samples}+\mathrm{Correctly}\;\mathrm{classified}\;\mathrm{samples}}\times 100\%$$

(9) $$\mathrm{Error}\;\mathrm{Rate}=\frac{\mathrm{Misclassified}\;\mathrm{samples}}{\mathrm{Misclassified}\;\mathrm{samples}+\mathrm{Correctly}\;\mathrm{classified}\;\mathrm{samples}}\times 100\%$$

The SOM model generated for the CoF measurement results based on the unsegmented force signals was analysed for correct classification (Figure 13a). The classification rate of the model in classifying a ‘high coefficient of friction’ is represented by the bars of 90% and 84.5%. The error rate in classifying samples designated as a ‘high coefficient of friction’ is represented by the bars of 10% and 15.5%. The error rate of the SOM classification of ‘low coefficient of friction’ was 0%. Finally, the classification rate of the model in classifying samples designated as a ‘low coefficient of friction’ is represented by the bars of 100%.

The classification results of the SOM model prepared based on the force signals subjected to the segmentation process are presented in Figure 13b. The classification rate of the model in classifying samples designated as a ‘high coefficient of friction’ is represented by the 99% and 98.5% bars. The error rate in classifying samples that exhibit a ‘high coefficient of friction’ is represented by the 1% and 1.5% bars. No errors were observed in classifying samples designated as a ‘low coefficient of friction’. The classification rate of the model in classifying samples from the ‘low coefficient of friction’ group is 100%.

Due to the use of an unsupervised classifier working with unbalanced data, the SOM indices (error rate and classification rate) were found to be highly satisfactory. The lower classification rate obtained for the ‘low coefficient of friction’ classification (Figure 13a,b) can be explained by the larger amount of data near the transition zone between the low and high coefficients of friction, which makes the classification more difficult. In Figure 12c, for high contact pressure, 50% of the obtained results are in the ‘low CoF’ range.

## 7. Summary and Conclusions

In this paper, a new approach is presented in the application of self-organising maps with unbalanced data for the classification of the CoF in terms of its value. A method for determining the CoF value using segmentation of force signals is proposed. Three input parameters of strip-drawing tests were used to validate the proposed method of processing force signals. The features that best describe the differences between low and high coefficients of friction were extracted based on statistical tests. The SOM obtained satisfactory results, with the CoF classification between 84.5% and 100% for unsegmented data, and between 98.5% and 100% for segmented data. The error rate values were between 0 and 15.5%, but better quality indicators were obtained for the SOM classifying ‘high coefficients of friction’. In the investigations, the two ranges of changes in the surface roughness parameter Sq were found. They were closely related to the changes in the CoF value.

The studies confirmed that the selected parameters and features of the strip-drawing test are able to classify the CoF in terms of its value based on the signals of force parameters. Moreover, it is possible to assess the CoF value without using expensive sensors or a combination of several different sensors.

It was also found that the proposed methodology and SOM model can be used as a CoF monitoring system, with a classification rate of up to 100%. The error rate, depending on the value of the classified CoF for segmented signals, was in the range of 0 to 1.5%. An unsupervised SOM working with unbalanced data was analysed, which allows for its implementation in an industrial environment. It is important to emphasise that the model should be trained for each specific strip-drawing test condition.

Production process control includes both supervision and management of the production process. In classic solutions, supervision of the production process is carried out by the operator in person, using conventional monitoring methods, sometimes supported by measuring devices. With the development of new technologies, supporting or replacing traditional methods of production process supervision with computerised and automated methods is becoming increasingly common. This allows for minimising the number of rejects, increasing repeatability, optimising processing conditions, and reducing failures and related downtimes in machine operation. The basic, primary function of the drawing process monitoring system is to determine the CoF. Coupling information on the CoF value with the results of measurements of the sheet metal’s Sq index allows, for example, for determining the level of wear of countersamples and the energy consumption of the process. Based on the CoF value, the drawing process monitoring system can indicate conditions that require correction (e.g., a sudden increase in forces or vibrations). In addition, documentation of the course of the operation allows for detecting human errors, such as a lack of lubricant, a change in its lubricating properties, or accidental changes in processing parameters.

Considering that only two commercial lubricants (S100 Plus and S300) were tested, we proposed considering in the future how the friction coefficient classification performance of the SOM model may differ when applied to new lubricants (e.g., nano-modified oils, bio-based lubricants, or water-based superlubricants).

The studies did not analyse friction-induced temperature changes in the contact zone. For lubricated sliding these are not a big issue, but for dry sliding, the friction can significantly change the contact conditions of the surface asperity peaks and change their mechanical properties. According to known physical laws, friction causes an increase in temperature in the contact zone; therefore, a quantitative analysis of temperature changes during strip-drawing tests will be the subject of future research.

## Figures and Tables

**Table 1:** Contents of alloying elements in steel DP780 (wt.%).

C	Mn	P	S	Si	Cr	Mo	Cu	Al	Fe
0.093	1.415	0.011	<0.002	0.2	0.057	0.103	0.052	0.067	balance

**Table 2:** Selected mechanical properties of DP780 steel sheets.

Specimen	Yield Stress R_p0.2_, MPa	Ultimate Tensile Stress R_m_, MPa	Elongation A_80_, %
1	531	830	21.4
2	528	827	20.2
3	532	831	19.1
4	518	825	18.7
5	526	826	20.0
Average value *:	527.0 (5.5)	827.7 (2.5)	19.9 (1.1)

*—The standard deviation is given in brackets.

**Table 3:** Basic surface roughness parameters of DP780 sheet metal in the as-received state.

Sq [µm]	Ssk	Sku	Sp [µm]	Sv [µm]	Sz [µm]	Sa [µm]
1.25	0.090	2.60	3.82	5.98	9.80	1.02

**Table 4:** Surface roughness measurement conditions.

Parameter	Description/Value
P-R-W profile filter	Dual Gaussian filter for calculating areal material ratio curve parameters with variably adjustable cut-offs (EN ISO 16610-21:2013 [37])
RK profile filter	Digital Gaussian filter with standard cut-off stages and with variably adjustable cut-offs (ISO EN 13565-1:1999 [38])
Cut-off wavelength λ_c_	0.8 mm
Cut-off wavelength λ_s_	0.0027 mm
Shortwave cut-off	300
Scanning speed	0.5 mm/s
Measured tip radius	5 μm

**Table 5:** Kinematic viscosity of the oils used in the tests.

Oil Type	Kinematic Viscosity at 40 °C, mm^2^/s
Deep-drawing oil S100 Plus (Naftochem, Kraków, Poland)	110
Deep-drawing oil S300 (Naftochem, Kraków, Poland)	360

**Table 6:** Parameters of the strip-drawing tests.

Test No	Kinematic Viscosity, mm^2^/s	Sliding Speed, mm/min	Nominal Contact Pressure, MPa
1	0 (Dry friction)	30	2
2			6
3			10
4			14
5		60	2
6			6
7			10
8			14
9		90	2
10			6
11			10
12			14
13	360 (Lubrication with S100 Plus oil)	30	2
14			6
15			10
16			14
17		60	2
18			6
19			10
20			14
21		90	2
22			6
23			10
24			14
25	1135 (Lubrication with S300 oil)	30	2
26			6
27			10
28			14
29		60	2
30			6
31			10
32			14
33		90	2
34			6
35			10
36			14

**Table 7:** Results of the probability *p* for the model taking into account the influence of friction process parameters on the value of CoF.

Parameter	*p*
Lubrication conditions	0.000000
Nominal pressure	0.001797
Sliding speed	0.959955

**Table 8:** Normalised CoF values.

Actual Value	Normalised Value
0.13	0
0.18	0.4
0.26	1

**Table 9:** Activation level values for individual network neurons.

Neuron	1-1	1-2	2-1	2-2
Activation value	0.255325	1.236187	1.084087	0.861116

**Table 10:** Numbers of cases assigned to individual neurons.

Set	Neurons			
	1-1	1-2	2-1	2-2
Train	5	8	7	6
Test	2	0	3	0
Validation	2	1	2	0

## Data Availability

The original contributions presented in this study are included in the article. Further inquiries can be directed to the corresponding author.
